# Power Consumption Modeling of Discontinuous Reception for Cellular Machine Type Communications

**DOI:** 10.3390/s19030617

**Published:** 2019-02-01

**Authors:** Yasir Mehmood, Lei Zhang, Anna Förster

**Affiliations:** 1 Sustainable Communication Networks Working Group, Faculty of Electrical Engineering, University of Bremen, 28359 Bremen, Germany; anna.foerster@comnets.uni-bremen.de; 2 Centre for Wireless Innovation, ECIT Institute, Queen’s University Belfast, Belfast BT3 9DT, UK

**Keywords:** machine-type communication, power-saving mechanisms, semi-Markov chain, power-saving factor, wake-up latency

## Abstract

Machine-type communication (MTC) is an emerging communication trend where intelligent machines are capable of communicating with each other without human intervention. Mobile cellular networks, with their wide range, high data rates, and continuously decreasing costs, offer a good infrastructure for implementing them. However, power consumption is a great issue, which has recently been addressed by 3GPP (3rd Generation Partnership Project) by defining power-saving mechanisms. In this paper, we address the problem of modeling these power-saving mechanisms. Currently existing modeling schemes do not consider the full range of states in the discontinuous reception (DRX) mechanism in LTE-A networks. We propose a semi-Markov based analytical model, which closes this gap and shows very good results in terms of predicting performance evaluation metrics, such as the power-saving factor and wake-up latency of MTC devices compared to simulation experiments. Furthermore, we offer an evaluation of the DRX parameters and their impact on power consumption of MTC devices.

## 1. Introduction

Machine-type communication (MTC), which is also termed as machine-to-machine (M2M), is realized as a communication style where two or more devices exchange data with each other or with a set of servers, mainly without human assistance. Therefore, MTC is also considered one of the potential enablers for supporting the Internet-of-Things (IoT) paradigm in the future. Moreover, MTC covers a vast range of applications which include intelligent transportation systems, security, smart metering and monitoring, logistical processes, warehouse management, e-heath monitoring, wildlife management, home automation, and many more [1,2].

Unlike those of traditional human-type communication (HTC), the features of MTC traffic are quite different from the legacy mobile traffic, such as voice and video in terms of the number of devices, their QoS requirements, device or chipset costs, and power consumption [3]. For instance, MTC traffic exhibits distinct traffic patterns based on the application area. Thus, the activity patterns in MTC also differ from those of traditional HTC traffic. In addition, some of the MTC applications generate packets on a regular basis, such as in the case of smart meters used for measuring consumption of electricity, gas, heat, and water. On the other hand, several MTC applications, such as critical e-health monitoring, security, industrial control, emergency alerting, fire alarms, and other priority monitoring alarms, generate event-based information resulting in the random nature of MTC traffic [4]. In addition, the number of MTC devices is expected to grow to 27 billion in 2024, according to Machina Research [5].

Several MTC applications, such as e-health, remote management and control, mobile computing, gaming, industrial wireless automation, and wildlife management, are growing vastly in popularity and receiving attention from service providers as well as end-consumers [6]. However, the majority of these MTC applications are driven by fixed-energy sources [7]. Therefore, these battery-driven applications sometimes need to be left unattended for a longer period of time, e.g., in areas where there are no direct power sources. Consequently, these MTC devices are expected to be energy-efficient in order to operate for a longer duration of time [8]. Therefore, lowering the power consumption in MTC networks is one of the major focuses of not only chipset manufacturers, but also telecom operators and service providers [3]. Addressing these aforementioned MTC requirements, The 3rd Generation Partnership Project (3GPP), a leading telecommunication standardization body, introduced a discontinuous reception (DRX) mechanism also in LTE/LTE-A networks. Therefore, the devices use only predefined intervals for performing an activity and remain silent otherwise in order to reduce their power consumption [9]. Consequently, the DRX mechanism potentially reduces the power consumption of a device at the cost of increased latency.

In order to estimate the power consumption of an MTC device in an LTE/LTE-A network, several efforts were made in the literature to analyze the performance of a DRX mechanism through analytical modeling. As a result, the power consumption of LTE/LTE-A devices was approximated, which can ultimately provide some insights regarding the expected operating time of the device. In the case of MTC devices, most of the existing power consumption models on DRX mechanism for regular HTC traffic may also be used for MTC applications generating regular traffic patterns. However, limited research efforts have been made to model the power consumption behavior of event-based MTC traffic. This is due to the distinct features of event-based MTC traffic, which make it quite crucial to analyze the characteristics of the DRX mechanism for estimating power consumption more precisely compared to its counterpart regular traffic [10]. One of the potential reasons is the contention-based random access, in which a huge number of devices may attempt several times to access the network, resulting in a high power consumption. Therefore, in the case of cellular MTCs, it is beneficial to model the DRX mechanism of LTE-A networks by considering the maximum possible parameters in order to estimate the device power consumption of random MTC traffic more precisely.

The remainder of the paper is organized as follows. Section 2 presents an overview of the DRX mechanism in an LTE-A network in order to introduce how it works and the related parameters. Section 3 covers the existing efforts made for the modeling and the analysis of the DRX mechanism. In addition, a detailed description of the proposed model for analyzing the DRX mechanism for MTC devices is given in Section 4. The descriptions of the simulation setup and parameters followed by the results are provided in Section 6. In the end, conclusions are drawn in Section 7.

## 2. Overview of DRX Mechanism

In an LTE-A network, when a device is powered on, it usually has two radio resource control (RRC) states, which include RRC_Connected and RRC_Idle. The transition from RRC_Connected to RRC_Idle and vice versa depends on the packet activity. An Inactivity timer is triggered to push the device from the RRC_Connected to the RRC_Idle state. When there is no traffic activity until the expiry of the inactivity timer, the device transitions from the RRC_Connected to the RRC_Idle state. Moreover, the RRC inactivity timer is reset with any uplink or downlink data activity [9].

**Figure 1:**
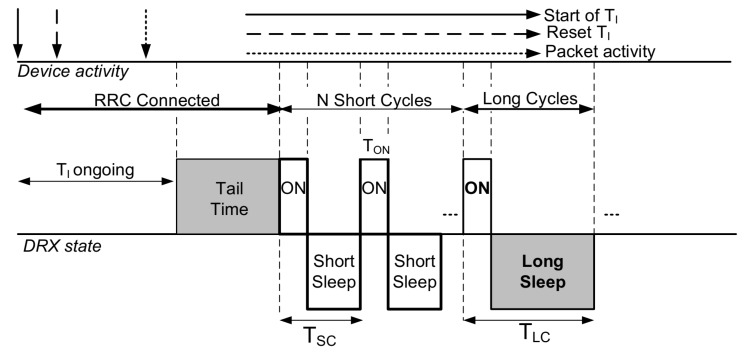
An overview of the discontinuous reception (DRX) mechanism in LTE-A.

In an LTE-A network, a device may be configured with a DRX mechanism by RRC in both connected and idle states. However, the functionality and configuration parameters in different states also vary. Since data activity mainly occurs in a connected state, the main target modes are in the active and DRX states. In addition to the RRC Inactivity timer, the network also triggers a DRX inactivity timer to move the device from active mode to DRX mode. When there is no packet activity, the device stays connected but transitions to the DRX mode. Furthermore, the DRX inactivity timer is reset when there is a packet activity [11]. In an LTE-A network, the RRC inactivity timer is set to be greater than the DRX Inactivity timer. An overview of the DRX mechanism in an LTE-A network is illustrated in Figure 1.

In an LTE-A network, the DRX mode is configured by several parameters and timers, such as the on duration timer $\left(T_{on}\right)$, DRX inactivity Timer $\left(T_{I}\right)$, short cycle timer $\left(T_{SC}\right)$, and long cycle timer $\left(T_{LC}\right)$, and optionally, the number of short cycles (N). $T_{I}$ is restarted on any downlink packet indication in PDCCH (physical downlink control channel) or uplink packet transmission. Upon the expiry of $T_{I}$, the device enters the short DRX cycle [12]. After *N* consecutive short DRX cycles, the device enters the long DRX cycle. If the short DRX cycle is not configured, the device enters the long DRX cycle directly. However, the short DRX cycle is important to be configured for delay-sensitive traffic as it reduces the overall latency and the wake-up delay. A brief description of various DRX parameters is provided in Table 1.

## 3. Literature Review

In the literature, several efforts were made to study the DRX mechanism in an LTE-A network. Moreover, these efforts were made in order to study the DRX mechanism for both MTC and HTC traffic by taking into account various parameters of DRX. For instance, the authors of [12] studied power consumption for active and background traffic, thus proposing two semi-Markov models. Furthermore, in the case of active traffic, each session consists of multiple packet calls, whereas each session in the case of background traffic mainly consists of one or two short packets. However, the proposed semi-Markov models are designed for modern mobile Internet data. In addition, the authors of [14] proposed an analytical model for studying power consumption in the case of MTC in an LTE-A network. Several parameters were taken into account, such as the DRX inactivity timer, short DRX cycle, long DRX cycle, etc. However, the RRC connection time and the RRC release time (also called tail time) were neglected in Reference [14]. In addition, the authors of [15] proposed an alternative DRX model for estimating power consumption in cellular MTC. Moreover, the authors introduced RRC active mode, RRC connection, and the release timer in their proposed model for analyzing the DRX mechanism. However, the expected arrivals during the RRC release period and possible power consumption variations during the active state were not taken into account. Further, in References [16,17,18], various DRX models were proposed for conventional HTC traffic, thus not directly applicable to MTC traffic. In addition, the authors of [19] considered multiple received packets within a short or a long sleep state, and thus estimated the average delay and power consumption for Poisson-based MTC traffic. Unlike in Reference [19], the multimedia traffic which is modeled as a heavy-tailed type I Pareto distribution was considered for modeling a DRX mechanism in Reference [20]. Moreover, the authors of [21] proposed a partially observable Markov decision process (POMDP) framework to configure the relationship between short and long cycles to maximize the energy-saving efficiency under the certain constraint of delay. Nevertheless, the RRC connection, tail time as well as the power consumption variations within the active mode were neglected in References [19,20,21].

In addition to the existing models given in Table 2 for analyzing a DRX mechanism, the authors of [22,23] proposed power consumption models for the universal mobile telecommunication systems (UMTS) user equipment (UE) with constant power consumption values, such as idle state (1W) and active connections (3W). In addition, the authors of [24] proposed a more differentiated model consisting of five states, namely standby (weak signal), standby (strong signal), ringing, talking, and attempting call, which were investigated in terms of minimum, maximum, and average power consumption. However, the authors ignored the existence of context related parameters, such as cell environment, user mobility, device type, etc.

In this paper, a semi-Markov chain model is proposed for modeling a DRX mechanism in MTC, which is different from the existing models as it not only takes into account the probability of packet arrivals during the tail time but also considers power consumption variations during active mode based on the system and context parameters in order to better estimate the power consumption in MTC applications. The short DRX cycle is proposed which can be used for delay-sensitive HTC and MTC traffic [4]. A summary of the latest research efforts for modeling a DRX mechanism based on distinct parameters is given in Table 2.

## 4. System Model

In the system model, we first describe the power consumption variations during the active and non-active modes. Afterwards, the proposed semi-Markov chain model is comprehensively explained by considering numerous DRX parameters in an LTE-A network. The list of the used symbols for modeling the DRX mechanism is provided in Table 3.

### 4.1. Power Consumption in Active/Non-Active Modes

**Figure 2:**
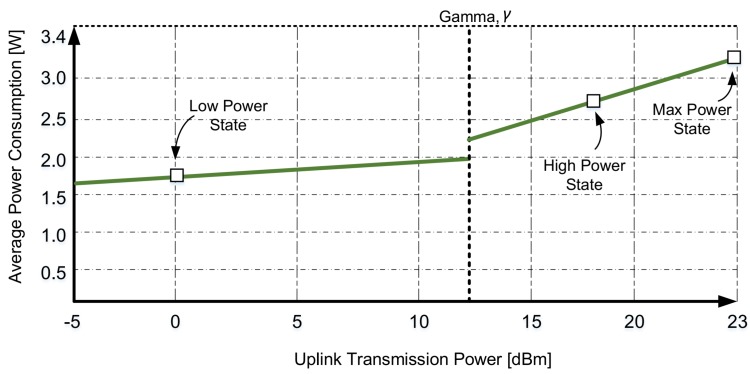
The power consumption curve, redrawn based on Reference [25].

According to Reference [25], the power consumption curve of a device can be separated into two regions based on its uplink transmission power $P_{TX}$ and the spatiotemporal variations of the radio channel, see Figure 2. Therefore, a power consumption threshold (γ) is set for separating the curve into two regions based on their slopes. The low transmission power is characterized by slope $\alpha_{L}$, whereas high power transmission is characterized by $\alpha_{H}$ in Reference [25]. In addition to slope parameters, $\beta_{L}$ and $\beta_{H}$ represent the y-intercept of the curve for the low and high power consumptions, respectively. Moreover, this particular characteristic can be observed for all devices. The main motivation behind this categorization is the different stages of the power amplifiers used within the devices. Therefore, power consumption, $P_{w}$ of a device can be independently approximated by the following linear functions:

(1) $$P_{w}=\left\{\begin{array}{c} \alpha_{L}\cdot P_{TX}+\beta_{L},\;\mathrm{if}\;P_{TX}\leq \gamma \\ \alpha_{H}\cdot P_{TX}+\beta_{H},\;\mathrm{if}\;P_{TX}>\gamma \end{array}\right..$$

Based on Equation (1), the empirical power consumption can be categorized into distinct modes. Therefore, a space of four distinct power states is introduced in this paper based on Reference [25]. Moreover, the power consumption values for the idle and the device transmission states were considered, which are described below.

In the non-active state, the device is not in a continuous reception mode. However, it can be in on duration, short sleep, long sleep, tail, or RRC connection state. Moreover, the power consumption is reduced to zero when the device is in short/long sleep state. However, in active state, the device is in a continuous reception mode, where it can transfer data. Since the power consumption curves were observed to be linear for all tested devices in Reference [25], the following power consumption states were considered within the active mode.

1. Low: In this state, the transmit power control (TPC) algorithm of LTE-A described in Reference [26] adjusts the transmission power in a way that a predefined target signal to noise ratio (SNR) at the eNB can be achieved. When the uplink transmission power $P_{TX}$ is below a device-specific threshold γ, $P_{TX}$ = 0 dBm was found out to be a typical value for the low power state [25]. Thus, the average power consumption in the low power state is ${\bar{P}}_{2}$ = $P_{w}$($P_{TX}$ = 0 dBm).
2. High: If the overall transmission power $P_{TX}$ is higher than the device-specific threshold γ, the device enters the high power state due to the use of different power amplifiers. Due to the linear behavior of power consumption in a high power state, as shown in Figure 2, the average power consumption in a high power state is set to be ${\bar{P}}_{3}=P_{w}\left(P_{TX}=\left(P_{max}+\gamma \right)/2\right)$.
3. Max: If the TPC algorithm can no longer achieve the target SNR by compensating for the path loss, the device enters the max power state, in which the device power consumption has reached the maximum value. Therefore, the average device power consumption for the max power state is selected to be ${\bar{P}}_{4}=P_{w}\left(P_{TX}=P_{max}\right)$.

### 4.2. Proposed Semi-Markov Model

**Figure 3:**
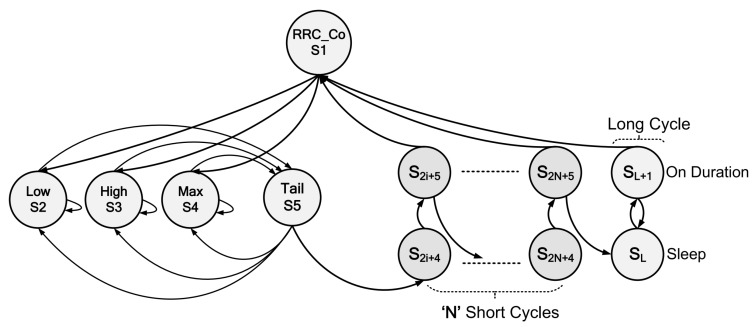
The proposed semi-Markov-based DRX model for MTC in LTE-A.

Since MTC covers a wide area of applications ranging from smart metering to emergency alerting, therefore, its traffic characteristics also vary. For instance, MTC devices mainly generate a huge traffic volume by accessing the network on a large scale. Therefore, the devices may attempt that several times to perform packet activities. Considering the random generation of MTC traffic, the input MTC traffic is modeled according to the Poisson process with a packet arrival rate of λ. Thus, the packet inter-arrival time, *t* follows an exponential distribution with a common mean value equal to 1/λ. The proposed semi-Markov chain model for the DRX mechanism for MTC devices is depicted in Figure 3. Moreover, the time spent in one particular state (holding time) is also considered a random variable, according to References [27,28]. In addition, the corresponding states in the semi-Markov chain model are described as follows:

1. $S_{1}$ represents the RRC connection setup state for the device to re-enter the network after releasing from *RRC_Connected*.
2. $S_{2}$, $S_{3}$, and $S_{4}$ correspond to the low, high, and max power consumption states as explained in Section 4.1.
3. $S_{5}$ is the RRC connection release state after the DRX inactivity timer expires and is called the tail time state [13].
4. $S_{6},S_{8},S_{10},\ldots ,S_{2N+4}$ are the *N* short sleep states within *N* short DRX cycles. The short sleep mode is proposed particularly for delay-sensitive MTC traffic.
5. $S_{7},S_{9},S_{11},\ldots ,S_{2N+5}$ are the *N* on duration periods within *N* short DRX cycles and therefore correspond to *N* short sleep states.
6. $S_{L}$ is the long sleep state within the long DRX cycle.
7. $S_{L+1}$ is the on duration period within the long DRX cycle, which corresponds to the long sleep state.

In the proposed semi-Markov model, parameters such as the DRX inactivity timer $T_{I}$, short DRX cycle $T_{SC}$, long DRX cycle $T_{LC}$, RRC release timer $T_{tail}$, and number of short cycles (*N*) are specified. In addition, the state transition probability from a state *j* to a state *k* in the proposed model is denoted by $p_{jk}$ with $j,k\in N^{+}$ and j,k∈{1,2,3,4,5,…,2N+4,2N+5,L,L+1}. Furthermore, the MTC device transmits and receives data in the active power modes, such as in states $S_{2}$, $S_{3}$, and $S_{4}$, and tail state $S_{5}$, as presented in Figure 3. The inactivity timer $T_{I}$ is restarted whenever there are uplink/downlink transmissions. Moreover, if there is no further data activity within the time period $T_{I}$, the device enters $S_{5}$. Otherwise, it enters into the corresponding active power state. Therefore, the respective transition probabilities for the above case are given in Equations (2) and (3):

(2) $$p_{22}=p_{33}=p_{44}=1-e^{-\lambda T_{I}},$$

(3) $$p_{25}=p_{35}=p_{45}=e^{-\lambda T_{I}}.$$

In state $S_{5}$, the device is still registered and attached to the network. Therefore, when a packet transmission/reception occurs during this state, the device directly enters into one of the corresponding active power states. Otherwise, the device is released from the network and thus enters into the short sleep cycles. The transition probabilities $p_{52}$, $p_{53}$, and $p_{54}$ for the above cases are given in Equations (4)–(6), respectively:

(4) $$p_{52}=\left(1-e^{-\lambda T_{tail}}\right)\theta_{2},$$

(5) $$p_{53}=\left(1-e^{-\lambda T_{tail}}\right)\theta_{3},$$

(6) $$p_{54}=\left(1-e^{-\lambda T_{tail}}\right)\theta_{4},$$

where $\theta_{2}$, $\theta_{3}$, and $\theta_{4}$ represent the probability of corresponding power states when the device is in a specific cell environment. It is further noted that $P_{TX}$ should remain updated according to the channel state information to ensure the packet activity during the tail state. In addition, in case of any uplink or downlink packet activity during the short sleep mode, the packets remain in buffer at the device or eNB, respectively, for the remaining duration of the short sleep period until the next corresponding on duration state. Therefore:

(7) $$p_{2i+4,2i+5}=1,$$

with $i\in N^{+}$ and i∈[1,N]. However, if there is no packet activity during the complete $i^{th}$ short cycle, the device then enters the ${\left(i+1\right)}^{th}$ short cycle. The corresponding transition probabilities are given in Equations (8) and (9):

(8) $$p_{2i+5,1}=1-e^{-\lambda T_{SC}}\;\mathrm{for}\;i\in \left[1,N\right],$$

(9) $$p_{2i+5,2i+6}=e^{-\lambda T_{SC}}\;\mathrm{for}\;i\in \left[1,N-1\right].$$

$T_{SC}$ represents the duration of a short cycle. If there is no packet activity during all *N* DRX short cycles, the device enters the long cycle. The respective transition probabilities are given in Equations (10)–(13):

(10) $$p_{L,L+1}=1,$$

(11) $$p_{2N+5,L}=e^{-\lambda T_{SC}},$$

(12) $$p_{L+1,1}=1-e^{-\lambda T_{LC}},$$

(13) $$p_{L+1,L}=e^{-\lambda T_{LC}}.$$

However, if the device has to wake up to perform any activity in order to send or receive data, it again establishes the RRC connection after being released from RRC_Connected state. Therefore, it goes through the RRC connection state from on duration in order to enter the active modes. The corresponding power state probabilities during active mode are $p_{12}=\theta_{2}$, $p_{13}=\theta_{3}$, and $p_{14}=\theta_{4}$. In addition, let $\pi_{j}$ denote the probability that the device stays in state $S_{j}$ during the steady state. Then, the following equations hold for the stationary probability distribution, as given in Equation (14):

(14) $$\left\{\begin{array}{c} \pi_{j}=\sum_{k=1}^{L+1}\pi_{k}\cdot p_{kj}..\;\mathrm{for}\;j\in \left\{1,2,3,4,\ldots L+1\right\}\\ \;\sum_{j=1}^{L+1}\pi_{j}=1 \end{array}\right..$$

Thus, we can obtain the stationary probability distributions which are given in Equation (15):

(15) $$\prod =\left\{\begin{array}{c} \pi_{1}=\frac{e^{-\lambda T_{tail}}}{K}\\ \pi_{2}=\frac{e^{-\lambda T_{I}}\theta_{2}}{K}\\ \pi_{3}=\frac{e^{-\lambda T_{I}}\theta_{3}}{K}\\ \pi_{4}=\frac{e^{-\lambda T_{I}}\theta_{4}}{K}\\ \pi_{5}=\frac{1}{K}\\ \pi_{2i+4}=\pi_{2i+5}=\frac{e^{-\lambda [T_{SC}\left(i-1\right)+T_{tail}]}}{K}\\ \pi_{L}=\pi_{L+1}=\frac{e^{-\lambda T_{tail}}e^{-\lambda NT_{SC}}\left(1-e^{-\lambda T_{LC}}\right)}{K} \end{array}\right.,$$

where *K* is denoted by:

(16) $$K=1+e^{-\lambda T_{tail}}+e^{-\lambda T_{I}}+2e^{-\lambda T_{tail}}\left[\frac{1-e^{-\lambda NT_{SC}}}{1-e^{-\lambda T_{SC}}}+\frac{e^{-\lambda NT_{SC}}}{1-e^{-\lambda T_{LC}}}\right].$$

Let $H_{j}$ denote the state holding time of state $S_{j}$, whereas $T_{tail}$, $T_{SC}$, $T_{LC}$, and the RRC Connection Time $T_{RC}$ are known, then the holding time can be expressed as $H_{5}=T_{tail}$, $H_{2N+4}=T_{SS}$, where $T_{SS}$ is defined as $T_{SC}-T_{ON}$, $H_{L}=T_{LS}$, where $T_{LS}=T_{LC}-T_{ON}$ and $H_{1}=T_{RC}$, respectively. Moreover, when the device is in the on duration state of the short DRX cycle, there can be three cases. For instance, a packet arrives after the expiry of the on duration timer with the probability $p_{2i+5,2i+6}$ or $p_{2N+5,L}$. In the second case, a packet arrives at the *l*th subframe of the on duration period with the probability of $p_{j}^{ON}$, which can be given as follows:

$$p_{j}^{ON}=e^{-\lambda (T_{SC}-T_{ON}+j-1)}-e^{-\lambda (T_{SC}-T_{ON}+j)},$$

whereas, in the third case, a packet arrives during the short sleep period with the probability $p_{s}=1-e^{-\lambda (T_{SC}-T_{ON})}$. Moreover, for the third case, we assume that the device enters the RRC connection state as soon as it enters the on duration state. Therefore, the state holding time will be $T_{sh}=0$. Thus, $H_{2i+5}$ can be given as follows:

(17) $$H_{2i+5}=p_{2i+5,2i+6}*T_{ON}+\sum_{j=1}^{T_{ON}}j*p_{j}^{ON}+T_{sh}*p_{s}=\frac{e^{-\lambda (T_{SC}-T_{ON})}-e^{-\lambda T_{SC}}}{1-e^{-\lambda }}.$$

On the other hand, when the device is within the on duration state of the long DRX cycle, there are also three cases. In the first case, the packet arrives after the expiry of the on duration with the probability $p_{L+1,L}$. In the second case, the packet arrives at the $j^{th}$ subframe of the on duration with probability $P_{j}^{LON}$. In the last case, the packet arrives during the long sleep period with probability $p_{LS}$. Therefore, $H_{L+1}$ is given as follows:

(18) $$H_{L+1}=p_{L+1,L}*T_{ON}+\sum_{j=1}^{T_{LON}}j*p_{j}^{LON}+T_{sh}*p_{LS}=\frac{e^{-\lambda (T_{LC}-T_{ON})}-e^{-\lambda T_{LC}}}{1-e^{-\lambda }}.$$

Additionally, when the device is within the active states, there are two cases. In the first case, the packet arrives after the expiry of the DRX inactivity timer with the probability $p_{25}$, $p_{35}$ or $p_{45}$, whereas, in the second case, the packet arrives at the $m^{th}$ subframe of the DRX inactivity timer with the probability $p_{m}=e^{-\lambda (m-1)}-e^{-\lambda m}$. Thus, $H_{2}$, $H_{3}$ and $H_{4}$ can be calculated as follows:

(19) $$H_{2}=H_{3}=H_{4}=T_{m}+\sum_{i=1}^{T_{m}}i*\frac{p_{i}}{p_{25}}=\frac{e^{-\lambda T_{m}}-1}{1-e^{-\lambda }}.$$

## 5. Performance Metrics

In this paper, we evaluate the performance of the proposed semi-Markov model based on metrics such as power-saving factor $\left(P_{S}\right)$ and wake-up latency (d). $P_{S}$ is defined as the percentage of time a device spends in sleep mode to reduce its power consumption. Hence, states $S_{6},S_{10},\ldots ,S_{2N+4}$ are the corresponding short sleep states, whereas the $S_{L+1}$ represents long sleep state, as given in Figure 3. $P_{S}$ can be calculated using Equation (20), whereas the expressions for $\pi_{i}$, $\pi_{L}$, $H_{i}$, and $H_{L}$ can be determined using Equations (15), (17) and (18).

(20) $$P_{S}=\frac{\sum_{i=1}^{N}\pi_{2i+4}H_{2i+4}+\pi_{L}H_{L}}{\sum_{i=1}^{L+1}\pi_{i}H_{i}}$$

The wake-up latency *d*, which is defined as the time interval between the packet arrival and departure, is usually experienced in the case of the RRC connection state and sleep modes. Furthermore, the packet arrival over the short DRX cycle and long DRX cycle follows, on average, a uniform distribution in the case of the Poisson arrival process. In the corresponding short and long DRX sleep states, the latency of short and long sleep modes can be interpreted as $d_{S}=\left(T_{SC}-T_{ON}\right)/2+T_{RC}$ and $d_{L}=\left(T_{LC}-T_{ON}\right)/2+T_{RC}$, respectively. Similarly, the latency values in the on duration state and RRC connection state are $T_{RC}$ and $T_{RC}/2$, respectively. Therefore, the wake-up Latency *d* can be calculated according to Equation (21):

(21) $$d=T_{RC}\left(\sum_{i=1}^{N}P_{2i+5}+P_{L+1}\right)+d_{S}\sum_{i=1}^{N}P_{2i+4}+d_{L}P_{L}+\frac{P_{1}T_{RC}}{2},$$

with $P_{j}=\frac{\pi_{j}H_{j}}{\sum_{j=1}^{L+1}\pi_{j}H_{j}}$.

## 6. Simulation Setup and Results Analysis

In order to validate the semi-Markov-based model proposed for analyzing a DRX mechanism in an LTE-A network, simulations were also performed using a simulation model developed in MATLAB. In the simulations, the packet arrival process was modeled according to the Poisson distribution. Thus, the packet inter-arrival time followed exponential distribution with common mean $\frac{1}{\lambda }$. In addition, the different stages of the DRX mechanism in LTE-A were also determined through several parameters, including timer values, transition probabilities related to various power states, as well as holding times. Furthermore, the packets arrived at different stages of the DRX mechanism based on the packet inter-arrival time. In the end, the related statistics were computed using the Monte Carlo method. Further, if not additionally specified at the simulation results, the used DRX simulation parameters and corresponding values are shown in Table 4. The DRX-related parameters used for the simulations were standardized in Reference [29], while the values of tail time $T_{tail}$ = 11.576 s and RRC connection time $T_{RC}$ = 260 ms were specified in Reference [30] based on the measurement results.

### Analytical Model Results Validation

In this subsection, the results obtained from the proposed analytical model for analyzing a DRX mechanism are validated through simulation results. For this reason, the performance metrics of the DRX mechanism, such as the power-saving factor and wake-up latency, were also determined via simulations by modeling all the DRX parameters.

**Figure 4:**
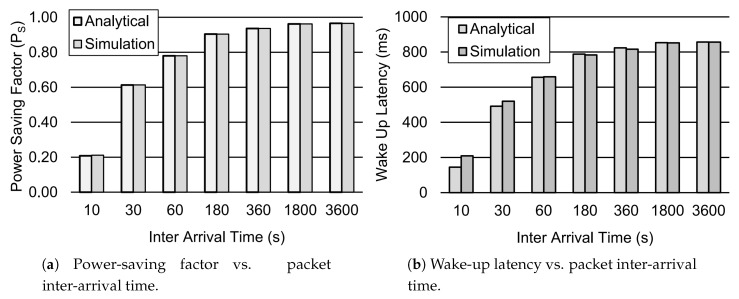
Performance analysis of the DRX mechanism for different values of packet inter-arrival time. Other parameter settings are $T_{I}=20$ ms, $T_{ON}=40$ ms, $T_{SC}=0.64$ s, $T_{LC}=1.28$ s, $T_{RC}=260$ ms, and $T_{tail}=11.576$ s.

Figure 4 shows how the power-saving factor $P_{S}$ and wake-up latency *d* vary according to the different values of packet inter-arrival time 1/λ. Therefore, Figure 4a shows the impact of packet inter-arrival time on $P_{S}$. Results showed that $P_{S}$ increases as the packet inter-arrival time increases. When the packet inter-arrival time is increased from 10s to 3600s, the $P_{S}$ factor is approximately increased by 79%. This means that the device remains in sleep mode more often for longer packet inter-arrival times. Similarly, the wake-up latency *d* also increases for the increasing packet inter-arrival time, as depicted in Figure 4b. The wake-up Latency is approximately increased by 83% when the packet inter-arrival time is raised from 10 s to 3600 s. This shows that the packets are buffered in the device for longer periods of time for greater values of packet inter-arrival time.

**Figure 5:**
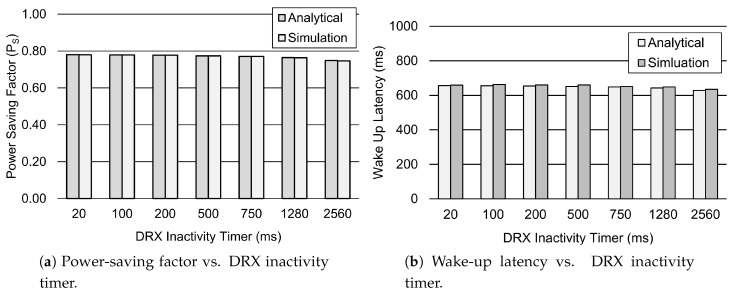
Performance analysis of the DRX mechanism for different values of $T_{I}$. Other parameter settings are $\frac{1}{\lambda }=60$ s, $T_{ON}=40$ ms, $T_{SC}=0.64$ s, $T_{LC}=1.28$ s, $T_{RC}=260$ ms, and $T_{tail}=11.576$ s.

Similarly, Figure 5 shows the impact of the DRX inactivity time $T_{I}$ on the power-saving factor $P_{S}$ and wake-up latency *d*. Results showed that the power-saving factor decreases gradually as the value of the DRX inactivity timer increases, see Figure 5a. When the value of the DRX inactivity timer increases from 20 ms to 2560 ms, the power-saving factor is approximately decreased by 5%. Consequently, this increases the device power consumption as it spends more time in active mode due to the longer DRX inactivity timer. Nevertheless, Figure 5b further shows that the wake-up latency is decreased by 4% for the increasing values of the DRX inactivity timer due to the given value of tail time $T_{tail}=11.576$ s, which increases the probability of a packet arrival during the tail state.

**Figure 6:**
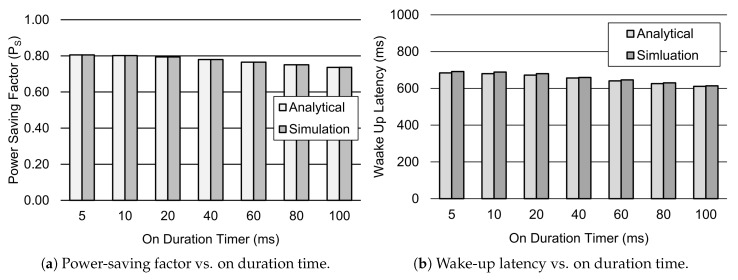
Performance analysis of the DRX mechanism for different values of $T_{ON}$. Other parameter settings include $\frac{1}{\lambda }=60$ s, $T_{I}=20$ ms, $T_{SC}=0.64$ s, $T_{LC}=1.28$ s, $T_{RC}=260$ ms, and $T_{tail}=11.576$ s.

Figure 6 presents the impact of the different values of the on duration Timer $T_{ON}$ on the power-saving factor $P_{S}$ and wake-up latency *d*. Figure 6a shows the impact of the on duration Timer on $P_{S}$. Results showed that $P_{S}$ decreases approximately 9% when the value of $T_{ON}$ is raised from 5 ms to 100 ms. This is due to the fact that the device consumes more power during on duration. Moreover, the wake-up latency *d* decreases by approximately 11% for the increasing values of $T_{ON}$, as shown in Figure 6b. Since the packet inter-arrival time is set to 60 s, $T_{ON}$ has a relatively small impact on the DRX mechanism.

**Figure 7:**
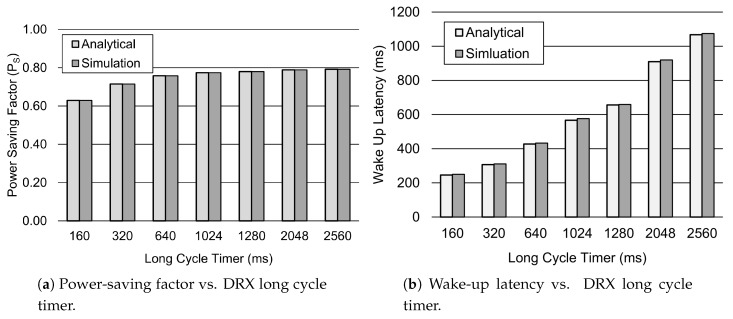
Performance analysis of the DRX mechanism for different values of $T_{LC}$. Other parameter settings include $\frac{1}{\lambda }=60$ s, $T_{I}=20$ ms, $T_{ON}=40$ ms, $T_{SC}=0.64$ s, $T_{RC}=260$ ms, and $T_{tail}=11.576$ s.

In Figure 7, the influence of various long cycle timers is investigated. The power-saving factor $P_{S}$ increases for the larger values of the long cycle timer due to the fact that the device spends more time in sleep mode, as depicted in Figure 7a. For instance, the $P_{S}$ factor increases approximately 20% when the long cycle timer value is increased from 160 ms to 2560 ms. As a result, the overall power consumption of the device decreases. Furthermore, Figure 7b shows that the wake-up latency *d* significantly increases for the larger values of the long cycle timer. For instance, when the long cycle timer value is raised to 2560 ms, the wake-up latency increases by 76%.

**Figure 8:**
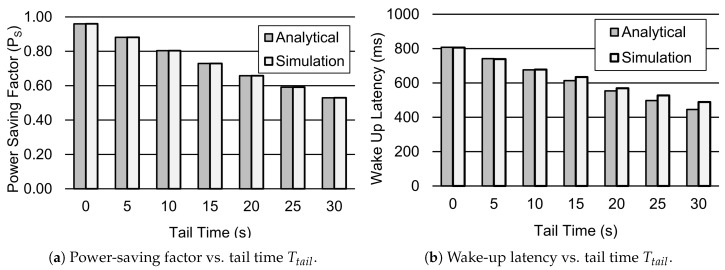
Performance analysis of the DRX mechanism for different values $T_{tail}$. Other parameter settings include $\frac{1}{\lambda }=60$ s, $T_{I}=20$ ms, $T_{ON}=40$ ms, $T_{SC}=0.64$ s, $T_{LC}=1.28$ s, $T_{RC}=260$ ms.

Figure 8 shows the influence of distinct values of Tail time $T_{tail}$, which is usually set by operators. It is the trade-off between the device power consumption and packet delay performance. We used $T_{tail}$ values ranging from 0 s to 30 s in this work, according to Reference [30]. Results in Figure 8a show that the power-saving factor is approximately reduced by 45% when the value of $T_{tail}$ is increased from 0 to 30 s. Similarly, the wake-up Latency also reduces for the increasing values of $T_{tail}$, as given in Figure 8b. For instance, when the value of $T_{tail}$ is raised to 30 s, the wake-up latency is reduced by around 44%. This means that higher values of tail time improve the delay performances. Since a device also consumes significant power within the period of $T_{tail}$, its value should be kept very low in order to reduce its power consumption.

**Figure 9:**
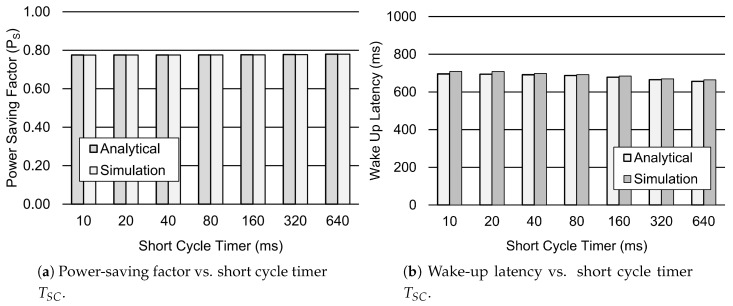
Performance analysis of the DRX mechanism for different values of $T_{SC}$. Other parameter settings include $\frac{1}{\lambda }=60$ s, $T_{I}=20$ ms, $T_{ON}=40$ ms, $N_{SC}=16$, $T_{LC}=1.28$ s, $T_{RC}=260$ ms, and $T_{tail}=11.576$ s.

**Figure 10:**
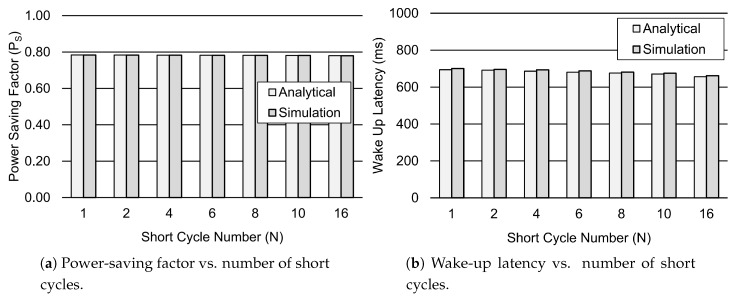
Performance analysis of the DRX mechanism for a different number of *N*. Other parameter settings include $\frac{1}{\lambda }=60$ s, $T_{I}=20$ ms, $T_{ON}=40$ ms, $T_{SC}=0.64$ s, $T_{LC}=1.28$ s, $T_{RC}=260$ ms, and $T_{tail}=11.576$ s.

Figure 9 depicts the influence of the short cycle timer on the performance metrics, such as the power-saving factor $P_{S}$ and wake-up latency. Figure 9a shows that the $P_{S}$ factor slightly increases for the larger values of $T_{SC}$. However, the wake-up Latency decreases for the increasing values of $T_{SC}$, as depicted in Figure 9b. This is due to the fact that the device is more likely to be in the short cycle before moving to the long cycle for the increasing values of $T_{SC}$. As a result, the wake-up latency decreases for the larger values of $T_{SC}$. Similarly, Figure 10 shows the impact of the number of short cycles on the power-saving factor and wake-up latency. Figure 10a shows that the $P_{S}$ factor decreases when the number of short cycles is increased. This is due to the fact that the device is less likely to go to the long cycle due to the greater number of short cycles. However, this improves the wake-up latency of the device when the number of short cycles is increased. Therefore, Figure 10b shows that the wake-up Latency reduces for the greater number of short cycles, such as 16.

From the aforementioned results, we found that the packet inter-arrival time has the highest impact on the power-saving factor as $P_{S}$ increases approximately by 79%. Furthermore, other DRX parameters such as the long cycle timer $T_{LC}$ and the tail time $T_{tail}$ also influence the $P_{S}$ factor significantly. Similarly, the wake-up latency is mainly influenced by the packet inter=arrival time and $T_{LC}$. Therefore, the wake-up Latency is increased by 83% and 76% when the values of packet inter=arrival time and the long cycle timer $T_{LC}$ are increased to 3600 s and 2560 ms, respectively. Furthermore, the tail time $T_{tail}$ affects the wake-up latency more than the on duration timer and short cycle timer.

## 7. Conclusions

In this paper, we proposed a semi-Markov chain model for analyzing the DRX mechanism in cellular MTC networks. The proposed analytical model was verified through simulation results. The performance metrics, such as the power-saving factor and wake-up Latency, were modeled with high precision and can be used for further research in the area of power saving for MTC communications, e.g., in Reference [31].

We further investigated the influence of various DRX-related parameters on the performance of the power-saving factor and wake-up latency. Our results showed that the packet inter-arrival time and the tail time influenced the $P_{S}$ factor the most. The $P_{S}$ factor was significantly increased and decreased for the greater values of packet inter-arrival time and the tail time, respectively. Moreover, the on duration and long cycle timers notably influenced the $P_{S}$ factor. On the other hand, the wake-up latency is maximum-influenced by the inter-arrival time and the on duration timer. However, the wake-up latency significantly decreases when the tail time increases.

In the future, the proposed semi-Markov model can also be adapted for modeling of the battery life time of a device. Consequently, the operating time of a device can be estimated with high precision.

## Figures and Tables

**Table 1:** List of various DRX parameters with definitions [9,13].

DRX Parameters	Definitions
Inactivity Timer $T_{I}$	No. of consecutive transmission time intervals (TTIs) for which a device decodes a PDCCH
On Duration Timer $T_{ON}$	No. of consecutive TTIs for which a device monitors PDCCH for radio resource allocation
Short Cycle Timer $T_{SC}$	No. of consecutive TTIs a device shall enter the short DRX cycle after $T_{I}$ expires
Short Cycle Number $N_{SC}$	No. of utilized Short DRX Cycle (optional)
Long Cycle Timer $T_{LC}$	No. of consecutive TTIs a device shall enter the long DRX cycle after short DRX cycle
RRC Connection Timer $T_{RC}$	No. of consecutive TTIs a device needs to reconnect to eNB after releasing from the network
Tail Timer $T_{tail}$	The time a device spends for transition from *RRC_Connected* to *RRC_Idle*

**Table 2:** A comparison of power consumption models based on the DRX mechanism. MTC: Machine-type communication; HTTP: HyperText Transfer Protocol; HTC: Human-type communication.

	*DRX Parameters*								
Ref. Paper	$T_{I}$	$T_{\mathrm{ON}}$	$T_{\mathrm{SC}}$	$T_{\mathrm{LC}}$	$T_{\mathrm{tail}}$	$T_{\mathrm{RC}}$	Arrivals in $T_{\mathrm{tail}}$	$P_{\mathrm{Active}}$ Variation	Traffic Type
[12] (Model 1)	**✓**	**✓**	**✓**	**✓**	**✗**	**✗**	**✗**	**✗**	Active
[12] (Model 2)	**✓**	**✓**	**✗**	**✓**	**✗**	**✗**	**✗**	**✗**	Background
[14]	**✓**	**✓**	**✓**	**✓**	**✗**	**✗**	**✗**	**✗**	MTC
[15]	**✓**	**✓**	**✗**	**✓**	**✓**	**✓**	**✗**	**✗**	MTC
[16]	**✓**	**✓**	**✓**	**✗**	**✗**	**✗**	**✗**	**✗**	HTTP
[17]	**✓**	**✓**	**✓**	**✓**	**✗**	**✗**	**✗**	**✗**	HTC
[18]	**✓**	**✗**	**✓**	**✓**	**✗**	**✗**	**✗**	**✗**	Bursty
[19]	**✓**	**✓**	**✓**	**✓**	**✗**	**✗**	**✗**	**✗**	Poisson
[20]	**✓**	**✓**	**✓**	**✓**	**✗**	**✗**	**✗**	**✗**	Multimedia
[21]	**✓**	**✓**	**✓**	**✓**	**✗**	**✗**	**✗**	**✗**	Poisson
Proposed Model	**✓**	**✓**	**✓**	**✓**	**✓**	**✓**	**✓**	**✓**	Poisson

**Table 3:** List of symbols used for modeling a DRX mechanism.

Symbol	Description
λ	Packet arrival rate in s^−1^
*T*	Packet inter-arrival time in s
α,β	Slope parameters for $P_{TX}$ line
$\theta_{k}$	Transition probability from RRC to active
$P_{w}$	Power consumption of device
$S_{i}$	State *i* where i∈[1,2,…,2N+5,L,L+1]
$p_{i,j}$	State transition probability from *i* to *j*
$\pi_{i}$	Stationary probability of state *i*
$H_{i}$	Holding time of state *i*
$P_{S}$	Power-saving factor
*d*	Wake-up latency

**Table 4:** List of DRX simulation parameters [29,30].

Simulation Parameters	Values
Packet inter-arrival time ($\frac{1}{\lambda }$)	60 s
Inactivity Timer ($T_{I}$)	20 ms
On Duration Timer ($T_{ON}$)	40 ms
Short Cycle Timer ($T_{SC}$)	640 ms
Short Cycle Number ($N_{SC}$)	16
Long Cycle Timer ($T_{LC}$)	1.28 s
RRC Connection Timer ($T_{RC}$)	260 ms
Tail Timer ($T_{tail}$)	11.576 s
Sample size	10

## Abbreviations

The following abbreviations are used in this manuscript:

3GPP	3rd Generation Partnership Project
DRX	Discontinous Reception
IoT	Internet-of-Things
HTC	Human-Type Communication
LTE	Long-Term Evolution
LTE-A	Long-Term Evolution Advanced
M2M	Machine-to-Machine
MTC	Machine-Type Communication
POMDP	Partially Observable Markov Decision Process
SNR	Signal to Noise Ratio
TPC	Transmit Power Control
UMTS	Universal Mobile Telecommunication System
